# The Incidence of Alopecia Areata in a COVID-19-Vaccinated Population: A Single-Center Review

**DOI:** 10.7759/cureus.50133

**Published:** 2023-12-07

**Authors:** Jeff Chen, Sebastian Cano-Besquet, Henrik Ghantarchyan, Michael M Neeki

**Affiliations:** 1 Internal Medicine, California University of Science and Medicine, Colton, USA; 2 Internal Medicine, Arrowhead Regional Medical Center, Colton, USA; 3 Emergency Medicine, Arrowhead Regional Medical Center, Colton, USA

**Keywords:** covid-19 vaccine, vaccination, covid-19, alopecia, alopecia areata

## Abstract

Background

Cases of alopecia areata (AA) attributed to the coronavirus disease 2019 (COVID-19) vaccination have been reported in recent literature. However, these reports are reflective of specific geographic areas, and whether this phenomenon is observed in other regions remains to be investigated. This study focused on the association between AA and COVID-19 vaccination among patients from a large single-center safety net hospital in California.

Methodology

In this study, using electronic health records of patients and publicly available vaccination data, the demographics of patients including age group, sex, and race along with the vaccination status were carefully reviewed.

Results

A total of 73 cases of AA in the period from the release of the COVID-19 vaccination on December 17, 2020, to February 10, 2023, were identified. The odds ratios (ORs) for developing AA among the vaccinated and unvaccinated for each demographic level were calculated. Among all vaccinated individuals, the OR for developing AA was 0.58 (95% confidence interval (CI) = 0.35-0.94, p-value = 0.02).

Conclusions

This investigation noted no apparent increase in the incidence of AA among the vaccinated population compared to the unvaccinated population.

## Introduction

Severe acute respiratory syndrome coronavirus 2 (SARS‑CoV‑2), a zoonotic virus, caused a major global pandemic outbreak and continues to pose a serious public health threat [1]. Several types of vaccines have been developed that aim to build protection by promoting the production of antibodies toward the spike protein antigen [2-4]. BNT162b2 (Pfizer) and mRNA-1273 (Moderna) belong to the mRNA vaccine family, while Ad26.COV2.S (Johnson & Johnson) is a modified and attenuated adenovirus that carries the DNA of the spike protein [2-4]. A recent study discussed a specific case of a patient diagnosed with pustular psoriasis a few days after coronavirus disease 2019 (COVID-19) vaccination and was treated successfully with an interleukin 1 antagonist medication [5]. The literature also found 28 other case reports reporting the development of pustular psoriasis after COVID-19 vaccination [5]. Similar to pustular psoriasis, alopecia areata (AA) is another autoimmune disorder.

Recently published reports from some parts of the world have reported the onset of AA in some individuals who received the COVID-19 vaccination [6-8]. Tassone and colleagues from Italy reported the occurrence of AA weeks after receiving mRNA or adenovirus COVID-19 vaccination in 24 patients [6]. Another case report from Italy reported an individual with rapid onset of AA after receiving the second dose of BNT162b2 (Pfizer) mRNA COVID-19 vaccine [7]. Furthermore, cases of AA have also been reported in Iran within two weeks of administration of AZD1222 (Oxford/AstraZeneca) COVID-19 vaccinations [8].

AA is a type of non-scarring hair loss and typically presents with well-circumscribed patches and exclamation-mark hairs, which are hairs with narrowed hair roots [9,10]. The incidence of AA is about 20 per 100,000 people annually and shows no gender predilection [9]. Although the incidence is relatively low, the disease has the potential to be debilitating, as hair loss can be a triggering factor for anxiety and depression [11,12]. As alopecia develops from the dysfunction of the suppression of major histocompatibility complex class I and the generation of inhibitory signals to immune cells, some triggers such as trauma, infection, and stress may precipitate the clinical presentation of AA in genetically susceptible individuals [10].

This study aims to investigate the correlation between AA occurrence and COVID-19 vaccination in a large safety net regional hospital in San Bernardino County (SBC).

## Materials and methods

Study design

This is a retrospective cohort study of patients with AA diagnosed at Arrowhead Regional Medical Center (ARMC) in Colton, CA at the demographic levels of age group, sex, and race. All cases of initial diagnosis of AA at ARMC occurred between December 17, 2020, and February 10, 2023. The study period was chosen to include the period from the initial COVID-19 vaccination administration to the time of data acquisition for this study. This study was approved by the institutional review board at ARMC (protocol #22-56).

Data sources and study population

The hospital-wide electronic health record (EHR) search for the initial diagnosis of AA using the International Classification of Diseases, Tenth Edition codes L63.0-L63.9 at ARMC was conducted. ARMC is a 456-bed, university-affiliated, teaching, public hospital that serves as a safety net hospital for the uninsured and underserved population in SBC, California. San Bernardino County is the largest geographic county in the United States, with a diverse population of 2.2 million people. According to the 2018 Census data, the racial makeup of SBC was 52.3% Latino, 29.8% White, and 8.0% African American [13]. The patients’ COVID-19 vaccination statuses at the time of AA diagnosis were then confirmed via a detailed review of their EHRs.

Public data for COVID-19 vaccination rates in San Bernardino County from the San Bernardino County COVID-19 Vaccination Program (SBCCVP) were used for the control group [14]. SBC vaccination data also contains age group, sex, and race parameters that allow them to serve as controls for statistical analysis at those levels.

Concerning COVID-19 vaccination rates at the demographic level of race, we made an additional assumption to distribute a small vaccinated group of unknown race. SBCCVP records estimate the total SBC population at 2,200,340, of which 1,186,520 are Latino, and 974,974 are non-Latino. Of the 1,396,420 vaccinated, 572,291 are identified as Latino, 785,283 as non-Latino, and 38,846 as unknown race, which represents 2.78% of the vaccinated population. We redistributed the unknown race group to the Latino and non-Latino groups according to their proportions as reported in the 2020 census (56.2% Latino vs. 43.8% non-Latino) before statistical analysis. The participants of unknown race from the AA group were also redistributed in the same fashion (three in the unvaccinated group and six in the vaccinated group of AA).

Outcomes and exposures

Patients are defined as those who received an initial diagnosis of AA between December 17, 2020, and February 10, 2023. At ARMC, the COVID-19 vaccines administered were BNT162b2 (Pfizer), mRNA-1273 (Moderna), and Ad26.COV2.S (Johnson & Johnson) once each vaccine entered the market. Patients from the control group were considered vaccinated if they received one or more doses of any Food and Drug Administration-approved COVID-19 vaccine before the time of initial diagnosis in the case of patients, and on or before February 10, 2023, in the case of the control group individuals [14].

Statistical analysis

A Fisher’s exact test using a two-tailed alternative hypothesis was performed for all patients as one group and separately at the levels of age group (<5, 5-11, 12-17, 18-49, 50-64, and 65+), sex, and race (non-Latino and Latino). We calculated odds ratios (ORs) of AA incidence associated with vaccine exposure, as well as 95% confidence intervals (CIs) for the ORs, and p-values for all patients and each group separately at each demographic level. Analyses were performed in R version 4.1.2 (2021-11-01) using the command “fisher.test().” For these 2 x 2 exposure and outcome cases, p-values were calculated using the hypergeometric distribution. The null hypothesis states that the OR is equal to 1. See the R documentation for “fisher.test()” for further information on the statistical methods used.

## Results

**Figure 1:**
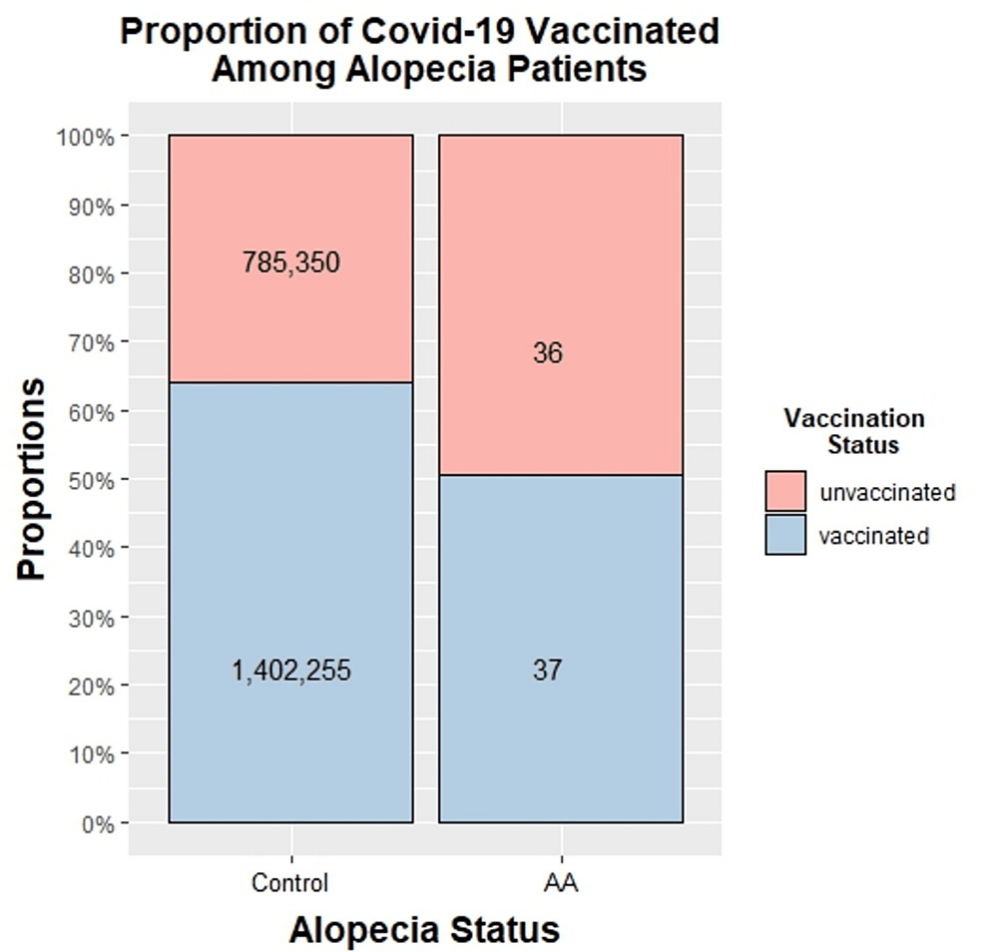
Percentages of those partially or fully vaccinated for COVID-19 among those with AA (“affected”) and the control group (“healthy individuals”) in the ARMC and SBC communities between December 17, 2020, and February 10, 2023. Counts are displayed for each of the categories (36 unvaccinated and 37 vaccinated in the AA group, and 785,350 unvaccinated and 1,402,255 vaccinated in the control group). AA = alopecia areata; ARMC = Arrowhead Regional Medical Center; COVID-19 = coronavirus disease 2019; SBC = San Bernardino County

**Figure 2:**
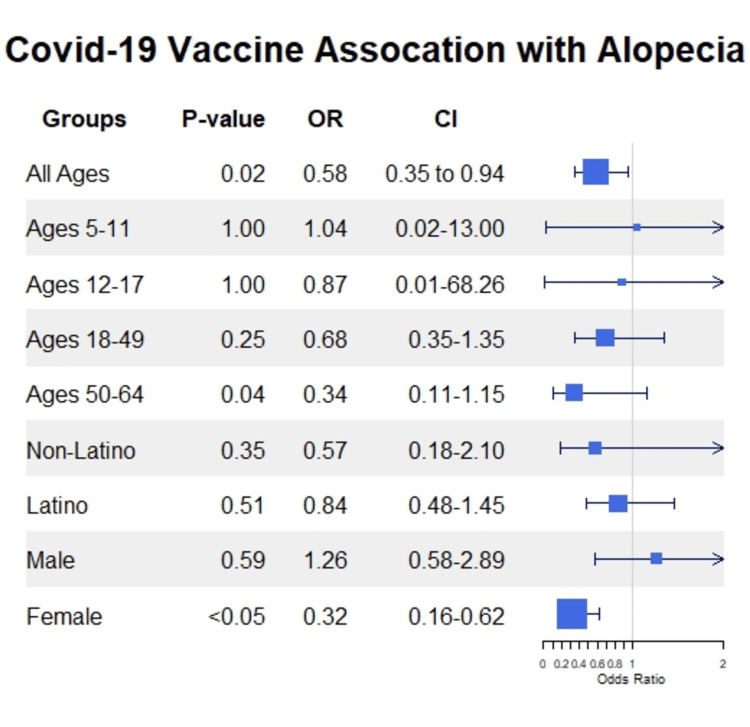
A forest tree plot showing the p-value, odds ratio (“OR”), and confidence interval (“CI”) for each demographic level (age, race, and race) in our patient cohort. Each square shows the OR for each demographic level, while the size of the square corresponds to precision. Blue line intervals and vectors indicate the 95% CI for each OR.

Between December 17, 2020, and February 10, 2023, 1,402,255 SBC residents had been either fully or partially vaccinated for COVID-19, while 785,250 had not received any vaccination. Over the same period, 73 patients at ARMC received an initial diagnosis of AA. Of those 73 patients, 36 had received no COVID-19 vaccination before the diagnosis of AA, while 37 had been either partially or fully vaccinated. The incidence of AA calculated with the provided 73 AA patients in a 1,402,255 population is roughly 0.0052%. Figure 1 is a stacked bar chart showing the relative proportions of those vaccinated among the AA and control groups and their respective counts (36 unvaccinated and 37 vaccinated in the AA group, and 785,350 unvaccinated and 1,402,255 vaccinated in the control group). Table 1 shows the number of individuals in each categorical group (unvaccinated vs. vaccinated). Figure 2 is a forest plot demonstrating the p-value, OR, and CI for the risk of AA at each population level (age group, sex, race). The probability of acquiring AA was lower in the vaccinated group overall, with an OR of 0.58 (95% CI = 0.35 to 0.94) and a p-value of 0.02 by Fisher’s exact test (see Figure 2). The risk of AA was also lower among the vaccinated females, with an OR of 0.32 (95% CI = 0.16 to 0.62) and a p-value of <0.05 (see Figure 2). At each demographic level, the risk of AA was either lower than the unvaccinated group or not statistically significant (see Figure 2).

**Table 1:** Number of individuals by sex, age, and race (Latino/non-Latino) in the alopecia areata and control groups. There are 73 patients in the alopecia areata group and 2,187,605 in the control group.

	Alopecia areata		Control croup	
	Not vaccinated	Vaccinated	Not vaccinated	Vaccinated (at least one dose)
Sex				
Male	11	21	434,609	660,104
Female	25	16	365,501	740,126
Age distribution				
All ages	36	37	799,384	1,400,956
<5	2	0	137,535	6,089
5–11	3	1	168,257	53,774
12–17	1	1	95,179	109,404
18–49	17	25	306,816	660,458
50–64	6	10	65,162	315,861
65+	7	0	26,435	255,370
Latino/Non-Latino				
Non-Latino	5	11	208,534	802,298
Latino	31	26	591,869	594,122

## Discussion

The result of this study is in line with Tassone et al.’s study, in which the authors reported that the number of AA cases before the COVID-19 pandemic was not statistically significantly different from the number of cases during the COVID-19 vaccination campaign [6]. Additional investigation suggested factors, such as gender, rather than the COVID-19 vaccine itself as a culprit to the reported cases of AA [6]. Tassone and colleagues reported 24 patients who developed AA after COVID-19 vaccination, 20 of whom were female, which yields a 5:1 gender ratio. This finding was larger than the nearly 2:1 ratio noted by Lundin and his team. Despite the difference in the ratios noted in the two above studies, both authors concluded that females are more likely to develop AA than males [15].

Although this study noted no clear association between COVID-19 vaccination and AA among the SBC population, a few studies reported otherwise [16,17]. Proposed mechanisms of AA induced by vaccination include the involvement of the RRARSVAS peptide (a surface glycoprotein) on the S protein produced by the BNT162b2 (Pfizer) and mRNA-1273 (Moderna) vaccines [16]. The RRARSVAS peptide is found to strongly bind to the HLA-B*27:05 allele, which is present in 6% of the population, and it seems to be associated with a low risk of autoimmunity [16]. The molecular mimicry mechanism discussed above and its involvement with genomic variations in individuals is suggested to be a potential mechanism for inducing AA in some who received COVID-19 vaccination [16]. An additional suggested mechanism is the molecular mimicry between SARS-CoV-2 infection and autoimmune disease [16]. A matching peptide sequence between 23 SARS-CoV-2 peptides and proteins from the human proteome, which is expressed ubiquitously, including in the hair follicles, might promote the loss of peripheral tolerance [16]. Furthermore, the bystander theory could play a possible role in the development of self-reactive T lymphocytes in a patient infected with SARS-CoV-2 [17].

The genetic predisposition of the population may also play a role in the incidence of AA, as noted by Ortiz-Ramírez and colleagues [18]. One candidate gene affecting the predisposition to AA is *HR* (*Hairless*) [18]. Variants of the *HR *gene, c.3215T > A p.(Val1072Glu), noted in a study of 30 AA patients in Mexico which is believed to produce a dysfunctional protein that plays a key role in the development of hair follicles and thus was considered a risk factor for causing AA [18]. This may be an element influencing the results seen in this study as the majority of the patients seen in the hospital and the general county hospital were Latino [13]. Interestingly, there have been studies exploring the risk of contracting COVID-19 in individuals with AA versus those without AA [19]. The authors concluded that individuals with AA have a slightly decreased risk of contracting COVID-19 [19], potentially due to the protective effect of increased levels of interferon-gamma among the AA patients, which downregulates angiotensin-converting enzyme 2 in the body, the SARS-CoV receptor [20].

While the lower rate of AA incidence among the vaccinated is reassuring from a standpoint of vaccine safety, investigators are still uncertain about the observed reported results. Either the vaccine has a protective effect against AA or there are confounding variables at play that may put some unvaccinated patients at a higher risk of developing AA. One possible causative mechanism might have to do with immune system modulation. An increase in regulatory T cells in response to the vaccine might mitigate autoimmune activity that would otherwise be destructive to follicles [21]. Niessl et al. reported that T-cell inhibitory receptors are upregulated in the acute phase of COVID-19 infection [21]. Another potential mechanism suggested for reduced alopecia incidence in vaccinated patients is a reduction in anxiety and stress caused by fear of infection or a feeling of lack of control; stress is known to act as a contributor to or inducer of AA [22]. Juarez-Rendon and colleagues reported that >23% of patients diagnosed with AA had an emotional event or an identity crisis before the onset of AA [22]. Alternatively, there may be confounding variables that collectively contribute to the lower incidence of AA among the COVID-19 vaccinated. Possible triggers, other than emotional stress, may include infections, toxins, food, heritability in first-degree relatives, and a difference in prevalence among different ethnic and racial groups [22]. An example of the association between environmental triggers and disease flare-ups post-vaccination was provided in a study where patients with lower vitamin D levels were more prone to psoriasis exacerbation after COVID-19 vaccination [23].

A few possible limitations of this study should be considered. First, the retrospective design may have introduced selection bias and limited the availability of comprehensive data, potentially affecting the generalizability of the results. Additionally, the sample size could have impacted the study’s power to detect subtle associations. Moreover, external factors, such as comorbidities, variability, and individual responses to the vaccine, may have influenced the outcomes. Nonetheless, this study contributes to the growing body of knowledge on the importance of COVID-19 vaccination efforts set forth worldwide.

## Conclusions

Evidence from this study does not support the prior reports of an association between AA and COVID-19 vaccination, as supported by the ORs and p-values calculated from this single-center study. Investigators should consider future investigations regarding these unparalleled reports.
